# An ensemble machine learning approach to predict postoperative mortality in older patients undergoing emergency surgery

**DOI:** 10.1186/s12877-023-03969-0

**Published:** 2023-05-02

**Authors:** Sang-Wook Lee, Eun-Ho Lee, In-Cheol Choi

**Affiliations:** 1 Department of Anesthesiology and Pain Medicine, Asan Medical Center, University of Ulsan College of Medicine, 88 Olympic-ro 43-gil, Seoul, 05505 Songpa-gu Republic of Korea; 2 University of Ulsan College of Medicine, 88 Olympic-ro 43-gil, Seoul, 05505 Songpa-gu Republic of Korea

**Keywords:** Preoperative frailty, Emergency surgery, Machine learning, Hospital frailty risk score, Operation frailty risk score, Postoperative mortality

## Abstract

**Background:**

Prediction of preoperative frailty risk in the emergency setting is a challenging issue because preoperative evaluation cannot be done sufficiently. In a previous study, the preoperative frailty risk prediction model used only diagnostic and operation codes for emergency surgery and found poor predictive performance. This study developed a preoperative frailty prediction model using machine learning techniques that can be used in various clinical settings with improved predictive performance.

**Methods:**

This is a national cohort study including 22,448 patients who were older than 75 years and visited the hospital for emergency surgery from the cohort of older patients among the retrieved sample from the Korean National Health Insurance Service. The diagnostic and operation codes were one-hot encoded and entered into the predictive model using the extreme gradient boosting (XGBoost) as a machine learning technique. The predictive performance of the model for postoperative 90-day mortality was compared with those of previous frailty evaluation tools such as Operation Frailty Risk Score (OFRS) and Hospital Frailty Risk Score (HFRS) using the receiver operating characteristic curve analysis.

**Results:**

The predictive performance of the XGBoost, OFRS, and HFRS for postoperative 90-day mortality was 0.840, 0.607, and 0.588 on a c-statistics basis, respectively.

**Conclusions:**

Using machine learning techniques, XGBoost to predict postoperative 90-day mortality, using diagnostic and operation codes, the prediction performance was improved significantly over the previous risk assessment models such as OFRS and HFRS.

**Supplementary Information:**

The online version contains supplementary material available at 10.1186/s12877-023-03969-0.

## Introduction

As the proportion of older patients undergoing surgery increases worldwide, the evaluation of preoperative frailty is increasingly important [1, 2]. Frailty refers to a clinical condition in which physiological reserve is reduced and vulnerable to daily stressors [3]. Especially, an innocuous stress factor such as surgery in frail older patients is associated with poor clinical outcomes [4]. Predicting the frailty risk in older patients before surgery is an increasingly critical issue [1, 4]. Unfortunately, no established method or widely accepted model is available for the assessment of preoperative frailty in surgical patients. In previous frailty studies, the Fried model and Rockwood model were used [5, 6]. Most of the previous frailty measurement tools were time-consuming and required a lot of clinical evaluation and examination [7–12]. For example, frailty measurement methods such as gait speed, handgrip strength, or surveys of patients with many checklists [7–14]. Therefore, it had many limitations to be applied clinically, such as the condition in bed-ridden patients or emergency surgery, where there is not enough time for preoperative assessment. In a study by Gilbert et al., in 2018, the Hospital Frailty Risk Score (HFRS) was proposed as a frailty measurement tool using diagnostic code information from older patients [15] who were hospitalized in the emergency room. Therefore, there were many limitations to use in surgical patients. Similarly, another study predicted the preoperative frailty risk in surgical patients from only diagnostic code and operation code information that can be automatically extracted based on electronic medical records (EMR) data [16, 17] and Operation Frailty Risk Score (OFRS) using HFRS scores calculated from ICD-10 diagnostic codes and operation risk groups classified into eight categories were suggested [16, 17]. However, variation in subjective operation code classification, and difficulties to apply in their models based on specific hospital data, limit its usefulness [16, 17].

Therefore, in this study, we tried to develop a widely applicable predictive model based on the national health insurance database, which contains a wider range of multi-institutional data. Moreover, our study improved the predictive performance using artificial intelligence technologies such as machine learning, which are widely used in medical research recently.

## Methods

This study was approved by our local institutional review board. Written informed consent was exempted due to the retrospectively collecting of the data. The study was conducted in accordance with relevant guidelines and regulations or declaration of Helsinki. Machine learning modeling in this study was conducted according to the guidelines entitled “Guidelines for Development and Reporting Machine Learning Predictive Models in Biomedical Research: A Multidisciplinary View” [18].

### Data extraction

**Fig. 1:**
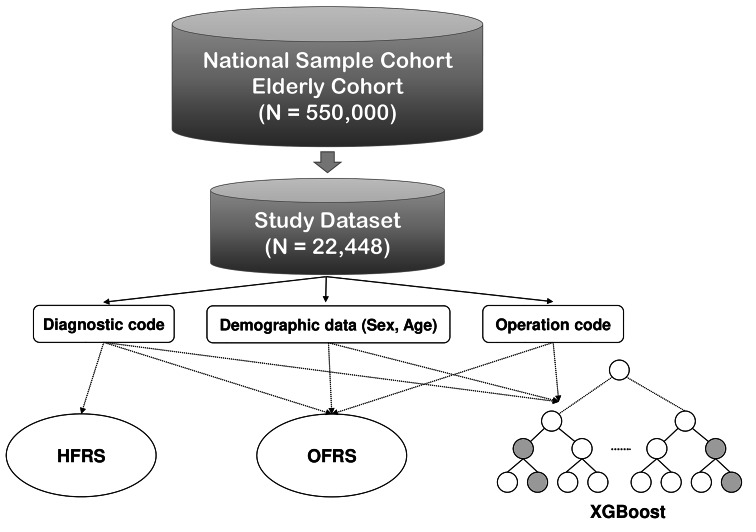
Flow chart showing the data retrieving procedures. HFRS: Hospital Frailty Risk Score, OFRS: Operation Frailty Risk Score

In this study, we retrieved the cohort data of older patients from the national sample cohorts (National Health Insurance Service – National Sample Cohort, version 2.0) provided by the Korean National Health Insurance Service (KNHIS). The cohort of older patients is a public health database of older patients (≥ 60 years old) from 2002 to 2013 [19]. The database covers insurance claim-related data and medical service-related data of approximately 550,000 older patients [19]. Among them, we extracted only 22,448 older patients (≥ 75 years old) who visited the hospital for emergency surgery (Fig. 1). Four variables such as age, sex, diagnosis code, and operation code, were used for analysis. Moreover, the national insurance claim codes were used for the operation code, and the ICD-10 code was used for the diagnostic code in which only the information recorded over the past year from the date of the surgery was extracted from the insurance database. The primary outcome of interest was 90-day postoperative mortality for the assessment tool of perioperative frailty. To obtain 90-day postoperative mortality, the data of all-cause deaths within 90 days after surgery were extracted from the death data in the KNHIS database. All data were downloaded only on the designated server computer according to KNHIS’ data policy, and all data analysis was conducted only on an allocated server.

### Hospital frailty risk score

The HFRS can be applied quickly to evaluate the frailty risk in the clinical field using ICD-10 diagnostic codes extracted from EMR [15]. In HFRS, 109 ICD-10 diagnostic codes for frailty were scored according to the advice from geriatric medicine experts. The HFRS was developed using cluster analysis in which the scores were given to ICD-10 codes that were at least twice as prevalent in a frail group compared to a non-frail group. In our study, HFRS was calculated based on ICD-10 codes diagnosed within one year from the date of surgery. Moreover, the HFRS is the total corresponding score if the diagnostic code matches 109 frailty-related codes suggested in the previous study [15].

### Operation frailty risk score

The OFRS was proposed in a recent study to evaluate preoperative frailty risk in surgical patients. Compared to HFRS, OFRS additionally used operation code information to reflect surgery-related risks. OFRS is the total points corresponding to the HFRS calculated from the diagnostic code, operation risk group for the operation code, and the patient’s age (Table S1) [16, 17]. Although the patient’s gender was originally considered in developing prediction model, it was excluded from the final scoring system because the effect of gender on outcome was not statistically significant (Table S1). According to the OFRS scoring system, age was divided into three categories, and HFRS as the total score calculated was divided by the three risk groups to give OFRS points to each group (Table S1) [16, 17]. Moreover, the operation-related insurance claim codes were classified into eight groups by clinical experts according to the risk of surgery (Table S2) [16, 17]. OFRS can obtain the final score by summing all the scores obtained in this way [16, 17]. The OFRS was classified as low risk if it was less than 2 points and high risk if it was greater than 4 points as described previously [16]. In this study, the total score was used in the analysis in the form of continuous variables instead of the risk groups.

### Machine learning modeling

A total of 772 operation codes and 98 diagnostic codes were converted into dummy variables, and each code was used in the model as an independent input variable. The missing values of the continuous variable were filled with the median values of the corresponding variable, but if the values of the categorical variable were missing, the data in corresponding row was excluded from the entire research dataset. We used the extreme gradient boosting (XGBoost) as a machine learning technique [20]. XGBoost is a boosting tree-based ensemble model that regressively improves the performance of the model to minimize the residuals for each iteration [20]. For model training, the entire dataset was randomly allocated, and 80% was used for training and the remaining 20% for the test. We use a 10-fold cross-validation technique, a method of dividing the training dataset into 10-fold datasets and then cross-validating each other to prevent the model overfitting. Hyperparameters were tuned for the best predictive performance in which the root mean square error is minimized by using bayesian optimization. The final hyperparameters tuned are as follows: max_depth was 6, min_child_weight was 1, gamma was 0.1, eta was 0.2 and subsample was 0.8. The area under the receiver operating characteristics (AUROC) curve was used to evaluate the predictive performance of the machine learning based prediction model and other prediction tools in the test dataset. The explainability of the model was enhanced by showing the feature importance plot to know the important variables in predicting the model’s outcome. The feature importance of the model was extracted based on three metrics of XGBoost algorithms: weight, cover, and gain.

### Statistical analysis

All data were analyzed only in the dedicated server space provided by KNHIS according to the policy on preventing the leakage of public data. Categorical variables are expressed as numbers and percentages, while continuous variables are expressed as means and standard deviations. The receiver operating characteristic curve analysis was used to compare the predictive performances of the prediction model using machine learning techniques and other risk scores. The significance level of statistical analysis was considered p < 0.05. All statistical analyses and machine learning modeling were performed with the R statistical language (R version 3·5·1, R Foundation for Statistical Computing, Vienna, Austria).

## Results

### Patients

The baseline characteristics of the studied patients according to the postoperative 90-day mortality rate are shown in Table 1. Of the total 22,448 patients, 3225 (14.4%) died within 90 days. The mean age of the patient group who died within 90-day post-operatively (death group) was higher than the no-death group (82.2 years vs. 81.0). Moreover, a significantly lower proportion of females was observed in the death group (50.2% vs. 62.2%, *p*-value < 0.001). Both HFRS and OFRS showed that the death group has higher points than the no-death group (Table 1). Subgroup analysis revealed a higher proportion of the death group in all high risk operation groups except in low risk operation groups 1 and 2 (*p*-value < 0.001).

**Table 1:** Patients’ characteristics classified by the presence of postoperative 90-day mortality

	Total	No	YES	*P*-value
*N*	22,448	19,223 (85.6%)	3225 (14.4%)	
Age, years	81.2 ± 4.9	81.0 ± 4.8	82.2 ± 5.4	< 0.001
Female	13,569 (60.4%)	11,950 (62.2%)	1619 (50.2%)	< 0.001
HFRS	6.2 ± 5.2	5.9 ± 5.1	7.6 ± 5.8	< 0.001
OP Group				< 0.001
Group 1	3906 (17.4%)	3724 (19.4%)	182 (5.6%)	
Group 2	5565 (24.8%)	5143 (26.8%)	422 (13.1%)	
Group 3	6940 (30.9%)	5597 (29.1%)	1343 (41.6%)	
Group 4	1660 (7.4%)	1358 (7.1%)	302 (9.4%)	
Group 5	3562 (15.9%)	2810 (14.6%)	752 (23.3%)	
Group 6	600 (2.7%)	434 (2.3%)	166 (5.1%)	
Group 7	94 (0.4%)	72 (0.4%)	22 (0.7%)	
Group 8	121 (0.5%)	85 (0.4%)	36 (1.1%)	
OFRS	3.2 ± 1.7	3.1 ± 1.7	3.8 ± 1.9	< 0.001

Data are presented as the mean ± standard deviation or number (percentage). HFRS: Hospital Frailty Risk Score, OP: operation, OFRS: Operation Frailty Risk Score

### Prediction performance of the models

**Fig. 2:**
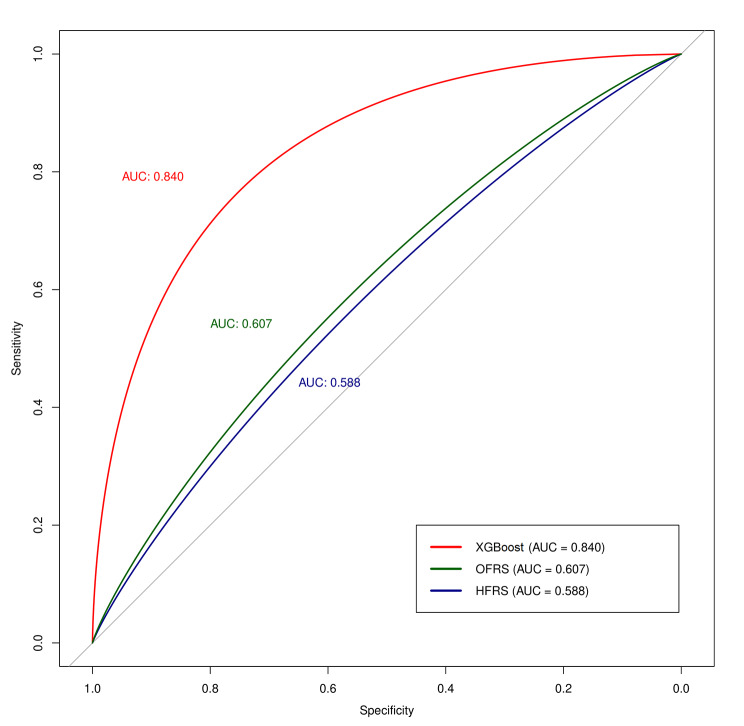
The performance of different predictive models by the receiver operating characteristic curve. XGBoost: Extreme Gradient Boosting, HFRS: Hospital Frailty Risk Score, OFRS: Operation Frailty Risk Score

The predictive performance of each prediction model for the postoperative 90-day mortality rate was compared with each other by the AUROC graph (Fig. 2). The prediction model using the XGBOOST algorithm showed the highest prediction performance at 0.840 on AUROC, while OFRS and HFRS showed relatively low prediction performance (AUROC of OFRS: 0.607, AUROC of HFRS: 0.588).

### Feature importance

**Fig. 3:**
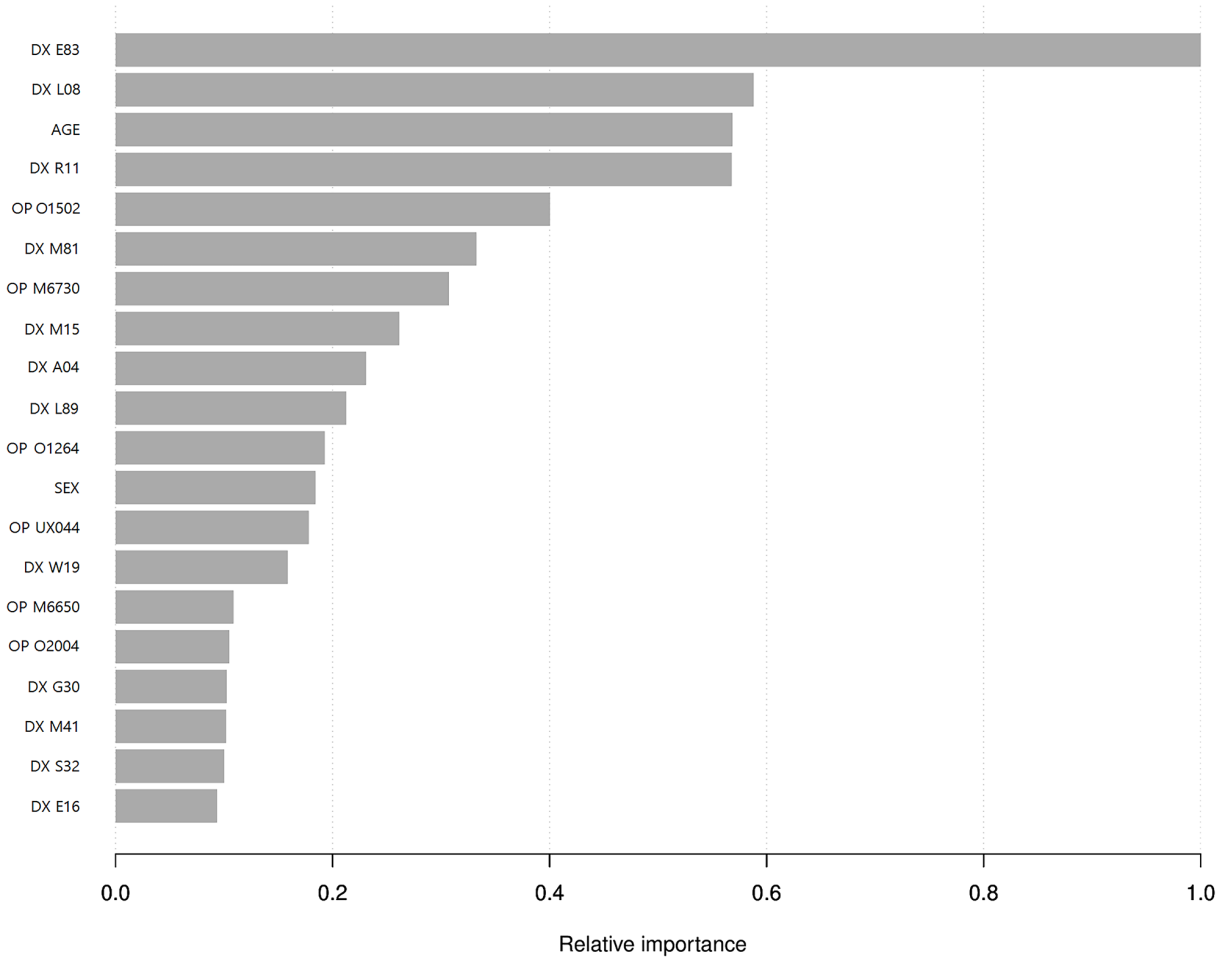
Feature of importance in the predictive model using machine learning method (XGBoost). XGBoost: Extreme Gradient Boosting, DX: Diagnostic code, OP: Operation code, E83: Disorders of mineral metabolism, L08: Other local infections of skin and subcutaneous tissue, R11: Nausea, and vomiting, O1502: Irrigation of empyema cavity, M81: Osteoporosis without current pathological fracture, M6730: Percutaneous gastrostomy, M15: Polyosteoarthritis, A04: Other bacterial intestinal infections, L89: Pressure ulcer, O1264: Operation of vocal cord paralysis, UX044: Temporomandibular joint arthrocentesis, W19: Unspecified fall, M6650: Percutaneous installation of inferior vena cava filter, O2004: Implantation of internal pulse generator by thoracotomy, G30: Alzheimer’s disease, M41L: Scoliosis, S32: Fracture of lumbar spine and pelvis, E16: Other disorders of pancreatic internal secretion

Figure 3 shows the feature importance of the XGBoost model. In addition to age, ICD-10 diagnostic codes A09 (Infectious gastroenteritis and colitis, unspecified), I95 (Hypotension), and L08 (Other local effects of skin and subcutaneous tissues) and operation code (O1502, Irrigation of empyema cavity) were shown as major factors affecting the postoperative 90-day mortality rate.

## Discussion

In this study, the predictive model made by learning operation code and diagnostic code using a machine learning technique called XGBoost had a better predictive performance for postoperative 90-day mortality, one of the indicators for preoperative frailty, compared to risk scoring systems such as OFRS and HFRS developed through conventional regression analysis.

Despite many attempts to evaluate and predict frailty in older patients in the last decades, a few frailty evaluation tools could be applied to older patients undergoing emergency surgery [16, 21]. Since most older patients undergoing emergency surgery are vulnerable to surgical stress and are likely to develop postoperative complications, predicting or evaluating the preoperative frailty of these patients clinically is very important [22]. To evaluate the frailty risk, most previous studies required interviews with patients or specific measurements such as grip strength or gait speed [7–14]. However, older patients who come to the hospital for emergency surgery are often restricted in communication, and many of them are bed-ridden conditions. Therefore, previous methods of frailty evaluation cannot be applied in clinical practice.

The previous study predicted the clinical outcome by measuring the frailty of hospitalized patients using ICD10 diagnostic codes related to frailty [15]. This was called HFRS, and the patient’s frailty was measured using an ICD10 diagnostic code that can be automatically extracted from EMR for inpatients who visited the emergency room [15]. However, since HFRS is a model designed for inpatients, there were many limitations to apply to the patients undergoing surgery. Therefore, recently, a study suggested a frailty prediction model, which was called OFRS that applied to surgical patients by additionally applying surgical code information [16, 17]. OFRS is a model that evaluates preoperative frailty using only the patient’s age, diagnostic code, and surgical code information [16, 17]. Therefore, OFRS has the advantage of being able to predict preoperative frailty without specific measurements or patient interviews in older patients undergoing emergency surgery. In the previous study, OFRS had poor predictive performance for clinical outcomes [16]. Previous work developed predictive models based on regression models, that is difficult to put a lot of operation code as input [16]. To overcome these, surgical codes were grouped according to similar risks, divided into the eight risk groups, and modeled for analysis[16] by the three clinical experts as they reviewed each other [16]. This type of grouping is not based on data, but the subjective views and experiences of clinicians [16]. Therefore, these subjective parts may lead to poor predictive performance, and these problems should be solved in a data-based or reliable objective manner [16]. In this study, to improve this low predictive power, we tried to overcome these limitations by utilizing an artificial intelligence model that has recently been applied in various medical fields.

We used an artificial intelligence technique, a tree-based ensemble model called XGBoost [20]. Existing tree-based machine learning methods are more useful in the medical field because they are similar to the traditional regression models that are commonly known and easier to find the cause for the results [23, 24]. The ensemble model refers to a technique that improves the final prediction performance by generating multiple trees and combining prediction results [20]. These machine learning techniques can be very useful for many input variables or the low incidences of the response variables [25]. To use statistical methods of existing traditional methods for modeling, the input variables must be grouped or the data must be artificially altered. However, the machine learning method works using the input without grouping the variables or data deterioration. Therefore, the prediction model of the machine learning method based on data is more accurate [25].

Moreover, our study was not based on the data retrieved from a single institution, but from a national public dataset. Therefore, this study presented a more robust predictive model compared to previous models based on specific institutions’ data. Furthermore, our work was based on a more generalized dataset and will serve as a cornerstone for creating predictive models applicable in many healthcare environments.

The prediction model using XGBoost showed higher prediction performance than OFRS or HFRS. This confirms that the predictive performance of the machine learning method’s modeling, which learns data as it is, is superior to the statistical modeling of the existing traditional method when there are many input variables as in this study. Using a machine learning technique, modeling is easy because there is no need to group surgical codes or process data. It may also be useful for application in changing clinical environments to update the predictive models using additional data. However, “black boxes” of the artificial intelligence are existed as to how artificial intelligence techniques work and why these results come out, are unable to be explained. Therefore, many techniques have recently been developed to overcome these limitations [26–28] identifying the main factors and causes of the predictive model with the model’s feature importance [27, 28].

Our study has many limitations. First, since it is a model using the sample cohort dataset provided by KNHIS, it is expected to be a more robust predictive model unlike previous studies, but it could not be confirmed without external validation with other institutional data. In future studies, external validation is needed. The second is that no comparative assessment was included on the diverse algorithms using other machine learning techniques such as random forest and support vector machine or deep learning methods other than the XGBoost. Moreover, according to the public data policy, data analysis could only be performed within the designated server provided by KNHIS, and it was difficult to apply the latest updates and algorithms of the designated server. Consequently, we were unable to conduct additional application and comparative research on various algorithms. Additionally, due to these policy-related limitations, it is currently difficult to publicly share and apply the developed prediction model in clinical settings. In the near future, it is expected that changes in data-related policies will enable the application of prediction models using public data in actual clinical fields. Another limitation of our study was that the predictive power of various clinical outcomes such as long hospital stay, readmission, extended intensive care unit stay, and reoperation other than postoperative mortality were not analyzed. Future studies were necessary to compare the performance of the prediction model presented in this study with other risk scores for more diverse clinical outcomes.

## Conclusion

In conclusion, by using machine learning techniques such as XGBoost to predict postoperative 90-day mortality, one of the indicators of preoperative frailty, using diagnostic and operation codes, the prediction performance improved over previous risks assessment models such as OFRS and HFRS. In the future, various artificial intelligence algorithms and external validation studies should be conducted.

## Electronic supplementary material

Below is the link to the electronic supplementary material.

Supplementary Material 1. Table S1. Scoring system for prediction of 90 day mortality. Table S2. Operation Groups according to surgical risk.

## Data Availability

All data regarding this study is available upon reasonable request to the corresponding author.
