# Supplementary material for: An ensemble machine learning approach to predict postoperative mortality in older patients undergoing emergency surgery
Source: BMC Geriatr. 2023 May 2;23:262. doi: 10.1186/s12877-023-03969-0 (PMC10155414; doi:10.1186/s12877-023-03969-0)
Supplement: Supplementary file 1 — Supplementary Material 1. Table S1. Scoring system for prediction of 90 day mortality. Table S2. Operation Groups according to surgical risk. [file 12877_2023_3969_MOESM1_ESM.docx]

**Supplementary Material**

***An ensemble machine learning approach to predict preoperative frailty in elderly patients undergoing emergency surgery***

Sang-Wook Lee, MD PhD^1^, Eun-Ho Lee, MD PhD^2^, In-Cheol Choi, MD PhD^1*^

**Supplementary Materials - Index**

| **Supplementary Tables** |  |
| --- | --- |
| **Table S1. Scoring system for prediction of 90 day mortality.** | *p3* |
| **Table S2. Operation Groups according to surgical risk.** | *p4* |
| **References** | *p23* |

**Table S1. Scoring system for prediction of 90 day mortality.**

| Variables | Categories | *Point* |
| --- | --- | --- |
| Age, years | 75 ~ 79 | 0 |
|  | 80 ~ 84 | 1 |
|  | 85 ~ 89 | 1 |
|  | ≥ 90 | 2 |
| Sex | Female | 0 |
|  | Male | 0 |
| HFRS | 0 | 0 |
|  | 1~4 | 1 |
|  | ≥ 5 | 2 |
| Operation Group | Group 1 | 0 |
|  | Group 2 | 1 |
|  | Group 3 | 0 |
|  | Group 4 | 2 |
|  | Group 5 | 4 |
|  | Group 6 | 4 |
|  | Group 7 | 4 |
|  | Group 8 | 6 |

1 point in scoring system reflect the increase in risk associated with a 10 year increase in age.

HFRS, hospital frailty risk score.

**Table S2. Operation Groups according to surgical risk.**

| **OG** | **Surgical depart** | **Operation code** | **Name of operation** |
| --- | --- | --- | --- |
| 1 | CS | O1318 | Endoscopic Cryotherapy[Tracheal, Bronchial, Lung Tumor] |
| 1 | DNT | U4430 | Alveoloplasty |
| 1 | DNT | U4690 | Reduction of Luxated Teeth |
| 1 | DNT | U4457 | Intraoral Antiphlogosis-Osteitis of Jaw, Osteomyelitis of Jaw etc. |
| 1 | ENT | O0951 | Single Nasal Polypectomy |
| 1 | ENT | S5572 | Foreign Body Removal of Ear Canal Or Removal of Impacted Cerumen-Extremely Complex |
| 1 | ENT | S5600 | Myringotomy |
| 1 | ENT | S5620 | Ventilation Tube Insertion |
| 1 | ENT | O1001 | Submucosal Resection or Septoplasty (Cartilage) |
| 1 | ENT | O1002 | Submucosal Resection or Septoplasty (Bone) |
| 1 | ENT | O1004 | Incision of Nasal Septal Hematoma or Abscess |
| 1 | ENT | O1010 | Inferior Turbinectomy |
| 1 | ENT | O1011 | Inferior Turbinectomy |
| 1 | ENT | O1015 | Turbinate Outfracture |
| 1 | ENT | O1050 | Intranasal Antrostomy |
| 1 | ENT | O1051 | Intranasal Antrostomy |
| 1 | ENT | O1092 | Operation of Frontal Sinus (Osteoplasty Following Brow Incision) |
| 1 | ENT | O1101 | Intranasal Ethmoidectomy |
| 1 | ENT | O1110 | Extranasal Ethmoidectomy |
| 1 | ENT | O1120 | Intranasal Sphenoidectomy |
| 1 | ENT | O1121 | Intranasal Sphenoidectomy |
| 1 | ENT | O1131 | Radical Operation of Pansinusitis |
| 1 | ENT | O1141 | Radical Operation of Maxillary And Ethmoid Sinus |
| 1 | ENT | O1152 | Radical Operation of Maxillary, Ethmoid And Sphenoid Sinus |
| 1 | ENT | O1161 | Radical Operation of Frontal And Ethmoid Sinus |
| 1 | ENT | O1176 | Radical Operation of Frontal, Ethmoid And Maxillary Sinus |
| 1 | ENT | O1215 | Removal of Epiglottic Cyst |
| 1 | ENT | O1231 | Removal of Vocal Nodule or Polyp |
| 1 | ENT | O1264 | Operation of Vocal Cord Paralysis, Foreign Material Injection (Unilateral) |
| 1 | ENT | OA273 | Operation of Laryngeal Stenosis, Laser Operation |
| 1 | ENT | Q2300 | Tonsillectomy |
| 1 | GS | M6700 | Removal of Biliary Residual Stone |
| 1 | GS | M6830 | Percutaneous Enterostomy |
| 1 | GS | M6850 | Cyst Aspiration |
| 1 | GS | Q7670 | Colonoscopic Removal Of Foreign Body |
| 1 | GS | M6721 | Fluoroscopic Dilatation of Upper Gastrointestinal Tract Stenosis with Balloon Catheter |
| 1 | GS | M6722 | Fluoroscopic Dilatation of Upper Gastrointestinal Tract Stenosis with Stent |
| 1 | GS | Q7692 | Colonoscopic Dilatation Of Colonic Stenosis-Stent Insertion |
| 1 | GS | Q7771 | Dilatation Of Bile Duct-Simple |
| 1 | GS | Q7773 | Removal Of Bile Duct Stone |
| 1 | GS | Q7774 | Removal Of Bile Duct Stone |
| 1 | GS | Q7701 | Colonoscopic Operation Of Colonic Tumor-Polypectomy |
| 1 | GS | Q7702 | Colonoscopic Operation Of Colonic Tumor |
| 1 | GS | N0900 | Excision of Ganglion |
| 1 | GS | P2121 | Operation For Axillary Lymph Node-Excision |
| 1 | GS | P2122 | Operation For Axillary Lymph Node-Dissection |
| 1 | GS | P2124 | Axillary Sentinel Lymph Node-Excision |
| 1 | GS | P2141 | Operation For Inguinal Lymph Node (Excision) |
| 1 | GS | P2142 | Operation For Inguinal Lymph Node (Dissection) |
| 1 | GS | Q3013 | Operation of Hemorrhoids-Hemorrhoidectomy |
| 1 | GS | Q7420 | Oddi's Sphincterotomy And Sphincteroplasty |
| 1 | GS | Q2936 | Perineal Operation-Others |
| 1 | GS | Q2977 | Seton Apply |
| 1 | GS | O0266 | Extensive Resection of Varicose Vein-Total Stripping of Saphenous Vein, Stab Abulsion of Varices |
| 1 | GY | R4521 | Dilatation And Curettage |
| 1 | GY | R4165 | Pelviscopic Fulguration |
| 1 | GY | R4240 | Polypectomy of Cervical Mucosa |
| 1 | GY | R4262 | Conization of Cervix-Loop Electrosurgical Excision |
| 1 | NS | N0471 | Percutaneous Vertebroplasty[Including Discography] |
| 1 | NS | N0472 | Percutaneous Vertebroplasty[Including Discography], From 2nd Site |
| 1 | NS | N0473 | Percutaneous Balloon Kyphoplasty[Including Discography] |
| 1 | NS | N0474 | Percutaneous Balloon Kyphoplasty[Including Discography] |
| 1 | NS | N1491 | Diskectomy (Invasive)-Cervical Spine |
| 1 | NS | N1492 | Diskectomy (Invasive)-Thoracic Spine |
| 1 | NS | N1493 | Diskectomy (Invasive)-Lumbar Spine |
| 1 | NS | N2472 | Removal of Implant For Internal Fixation of Spine[Posterior] |
| 1 | NS | S4595 | Neuroplasty-Major Peripheral Plexus |
| 1 | NS | S4596 | Neuroplasty-Hand And Foot |
| 1 | NS | S4825 | Radiofrequency Ablation of Spine, Percutaneous-Simple |
| 1 | NS | S4843 | Implantation of Intrathecal Drug Infusion Pump |
| 1 | NS | SY622 | Implantation, Change Or Removal of Spinal Neurostimulator Electrodes |
| 1 | NS | SY637 | Lead Implantation of Spinal Cord Stimulator And Trial Stimulation |
| 1 | NS | SY639 | Removal of Spinal Cord Stimulator Lead |
| 1 | NS | S4851 | Navigational Procedure for Surgery -> group 1 |
| 1 | OPH | S4880 | Evisceration |
| 1 | OPH | S4891 | Removal of Intraocular Foreign Body-With Magnet |
| 1 | OPH | S4895 | Removal of Intraorbital Foreign Body |
| 1 | OPH | S4911 | Implantation of Ocular Implant |
| 1 | OPH | S4921 | Surgery of Corneal Ulcer-Diathermy |
| 1 | OPH | S4923 | Surgery of Corneal Ulcer-Conjunctival Flap |
| 1 | OPH | S4941 | Conjunctival Suture |
| 1 | OPH | S4950 | Excision of Conjunctival Mass |
| 1 | OPH | S4960 | Curettage of Conjunctival Concretion |
| 1 | OPH | S4990 | Removal of Scleral Foreign Body |
| 1 | OPH | S5000 | Scleral Suture |
| 1 | OPH | S5011 | Scleral Transplantation |
| 1 | OPH | S5021 | Synechiolysis |
| 1 | OPH | S5030 | Optical Iridectomy |
| 1 | OPH | S5033 | Surgery for Glaucoma-Trabeculectomy |
| 1 | OPH | S5043 | Surgery for Glaucoma-Trabeculectomy |
| 1 | OPH | S5044 | Surgery for Glaucoma-Photocoagulation for Iris, Ciliary Body |
| 1 | OPH | S5049 | Glaucoma Implant Surgery |
| 1 | OPH | S5050 | Vitreous Aspiration |
| 1 | OPH | S5091 | Anterior Chamber Irrigation |
| 1 | OPH | S5111 | Surgery for Cataract Or Lens-Extracapsular Or Intracapsular Extraction |
| 1 | OPH | S5112 | Surgery for After Cataract |
| 1 | OPH | S5116 | Intraocular Lens Implantation-Secondary |
| 1 | OPH | S5117 | Intraocular Lens Implantation-Primary |
| 1 | OPH | S5118 | Intraocular Lens Exchange |
| 1 | OPH | S5119 | Surgery for Cataract Or Lens-Phacoemulsification |
| 1 | OPH | S5121 | Vitrectomy-Total |
| 1 | OPH | S5122 | Vitrectomy-Partial |
| 1 | OPH | S5130 | Retinal Detachment Surgery |
| 1 | OPH | S5140 | Cryopexy for Retinal Tear |
| 1 | OPH | S5145 | Periretinal Membrane Peeling |
| 1 | OPH | S5160 | Panretinal Photocoagulation |
| 1 | OPH | S5161 | Endolaser Photocoagulation |
| 1 | OPH | S5176 | Strabismus Surgery (Complex)-Multiple Muscle |
| 1 | OPH | S5231 | Removal of Orbital Tumor-Simple |
| 1 | OPH | S5245 | Excision of Eyelid Tumor-Benign |
| 1 | OPH | S5292 | Surgery for Blepharoptosis-Muscle Resection |
| 1 | OPH | S5293 | Surgery for Blepharoptosis-Others |
| 1 | OPH | S5321 | Conjunctival Sac Reformation-Partial |
| 1 | OPH | S5342 | Operation of Pterygium-Others |
| 1 | OPH | S5371 | Keratoplasty-Superficial |
| 1 | OPH | S5372 | Keratoplasty-Full Thickness |
| 1 | OPH | S5374 | Keratoplasty-Endothelial Lamellar |
| 1 | OPH | S5380 | Primary Closure of Cornea |
| 1 | OPH | S5421 | Suture of Eyelid Laceration-Simple |
| 1 | OPH | S5422 | Suture of Eyelid Laceration-Through And Through |
| 1 | OPH | S5450 | Tarsorrhaphy |
| 1 | OPH | S5480 | Reconstruction of Lacrimal Canaliculi |
| 1 | OPH | S5540 | Dacryocystorhinostomy |
| 1 | OS | M0031 | Removal of Foreign Body In Skin, Subcutaneous Tissue or Muscle with Incision of Fascia |
| 1 | OS | M0032 | Removal of Foreign Body In Skin, Subcutaneous Tissue or Muscle, Others |
| 1 | OS | N0641 | Closed Reduction of Fractured Extremity[Pelvis,Femur] |
| 1 | OS | N0642 | Closed Reduction of Fractured Extremity[Humerus,Tibia] |
| 1 | OS | N0643 | Closed Reduction of Fractured Extremity[Forearm Bone] |
| 1 | OS | N0644 | Closed Reduction of Fractured Extremity[Carpal Bone,Tarsal Bone] |
| 1 | OS | N0645 | Closed Reduction of Fractured Extremity[Metacarpal,Metatarsal,Finger,Toe] |
| 1 | OS | N0761 | Closed Reduction of Dislocation[Hip] |
| 1 | OS | N0762 | Closed Reduction of Dislocation[Shoulder] |
| 1 | OS | N0763 | Closed Reduction of Dislocation[Elbow,Knee] |
| 1 | OS | N0764 | Closed Reduction of Dislocation[Wrist,Ankle,Finger,Toe] |
| 1 | OS | N0780 | Brisement Force (Manipulation of Joint) |
| 1 | OS | N0912 | Simple Tendon Suture |
| 1 | OS | N0972 | Removal of Implant for Internal Fixation of Extremities[Femur] |
| 1 | OS | N0973 | Removal of Implant for Internal Fixation of Extremities[Humerus,Scapula] |
| 1 | OS | N0974 | Removal of Implant for Internal Fixation of Extremities[Radius And Ulnar, Tibia And Fibula] |
| 1 | OS | N0977 | Removal of Implant for Internal Fixation of Extremities[Radius and Ulnar, Tibia and Fibula] |
| 1 | OS | N0991 | Closed Pinning[Femur] |
| 1 | OS | N0995 | Closed Pinning[Clavicle,Patella,Carpal Bone,Tarsal Bone] |
| 1 | PS | N0210 | Operation For Ingrowing Nail |
| 1 | PS | N0215 | Guttering For Ingrowing Nail |
| 1 | PS | N0141 | Excision of Skin Benign Tumor (Simple,Superficial) |
| 1 | PS | N0142 | Excision of Skin Benign Tumor (Others,Extened To Muscle Layer) |
| 1 | PS | N0170 | Split Thickness Skin Graft-Others (900㎠ Over) |
| 1 | PS | N0173 | Split Thickness Skin Graft-Face or Joint (25㎠ Below) |
| 1 | PS | N0174 | Split Thickness Skin Graft-Face or Joint (25㎠ Over) |
| 1 | PS | N0175 | Split Thickness Skin Graft-Others (25㎠ Below) |
| 1 | PS | N0176 | Split Thickness Skin Graft-Others (25㎠∼100㎠) |
| 1 | PS | N0178 | Split Thickness Skin Graft-Others (100㎠∼400㎠) |
| 1 | PS | N0179 | Split Thickness Skin Graft-Others (400㎠∼900㎠) |
| 1 | PS | N0249 | Release of Scar Contracture And Flap Operation |
| 1 | PS | N0353 | Closed Reduction of Nasal Bone Fracture |
| 1 | PS | S0161 | Skin Flap-Local-Others |
| 1 | PS | S0164 | Island Flap-Others |
| 1 | PS | S0171 | Full Thickness Skin Graft (Face)-25㎠ Below |
| 1 | PS | S0173 | Full Thickness Skin Graft (Extremities)-25㎠ Below |
| 1 | PS | S0175 | Full Thickness Skin Graft (Others)-25㎠ Below |
| 1 | PS | S0176 | Full Thickness Skin Graft (Others)-25㎠ Over |
| 1 | PS | SA161 | Free Flap-Others |
| 1 | PS | SA164 | Free Comopsite Tissue Flap-Free Musculocutaneous Flap-Others |
| 1 | PS | SA165 | Free Omental Flap, Free Jejunal Flap-Others |
| 1 | PS | SB161 | Skin Flap-Local-Face |
| 1 | PS | SB165 | Muscle Flap-Face |
| 1 | PS | SB166 | Musculocutaneous Flap or Myocutaneous Flap-Face |
| 1 | PS | SB168 | Fasciocutaneous Flap-Face |
| 1 | PS | SB174 | Cadaveric Skin Graft (Others)-25㎠~100㎠ |
| 1 | PS | SC161 | Free Flap-Face |
| 1 | PS | SC163 | Free Composite Tissue Flap-Free Tendocutaneous Flap-Face |
| 1 | PS | SC164 | Free Composite Tissue Flap-Free Musculocutaneous Flap-Face |
| 1 | PS | N0153 | Mohs Micrographicsurgery |
| 1 | URO | R3191 | Transurethral Ureteral Dilatation-With Balloon |
| 1 | URO | R3211 | Percutaneous Ureterolithotomy |
| 1 | URO | R3264 | Ureteral Stent Indwelling-Operative |
| 1 | URO | R3267 | Removal of Ureteral Stent-Cystoscopic |
| 1 | URO | R3424 | Flexible Ureterorenoscopic Stone Removal-Kidney |
| 1 | URO | R3426 | Flexible Ureterorenoscopic Stone Removal-Ureter (Middle) |
| 1 | URO | R3563 | Operation For Urinary Incontinence-Foreign Material Or Autologous Fat Injection |
| 1 | URO | R3565 | Operation For Urinary Incontinence-Transvaginal Approach |
| 1 | URO | R3566 | Operation of Artificial Urethral Sphincter-Removal of Artificial Urethral Sphincter |
| 1 | URO | R3567 | Operation of Artificial Urethral Sphincter-Insertion of artificial urethral sphincter |
| 1 | URO | R3663 | Urethroscopic Surgery-Urethral Stent Indwelling |
| 1 | URO | R3665 | Urethroscopic Surgery-Urethrotomy |
| 1 | URO | R4060 | Excision of Batholin'S Gland And Cyst |
| 1 | URO | RA166 | Urinary Fistulectomy-Between Bladder And Intestine |
| 1 | URO | R3416 | Percutaneous Pyelostomy |
| 1 | URO | R3216 | Ureteroscopic Ureterolithotomy-Upper |
| 1 | URO | R3218 | Ureteroscopic Ureterolithotomy-Lower |
| 1 | URO | R3381 | Renal Pelvis Instillation-By Ureteral Catheterization |
| 1 | URO | R3440 | Ureterocutaneostomy |
| 1 | URO | R3541 | Transurethral Bladder Surgery-Tumor (Simple) |
| 1 | GS | Q7680 | Colonoscopic Bleeding Control |
| 1 | GS | Q7730 | Sigmoidoscopic Bleeding Control |
| 1 | GS | Q7752 | Sigmoidoscopic Operation Of Rectosigmoidal Tumor-Mucosal Resection And Submucosal Resection |
| 1 | GS | QX706 | Colonoscopic Operation of Colonic Tumor-Submucosal Dissection |
| 1 | GS | N7133 | Mastectomy-Benign (Partial) |
| 1 | GS | N7137 | Mastectomy-Malignant (Partial) |
| 1 | GS | P4551 | Total Thyroidectomy-Unilateral |
| 1 | GS | P4553 | Subtotal Thyroidectomy-Unilateral |
| 1 | GS | Q2755 | Operation of Inguinal Hernia (Others)-High Ligation |
| 1 | GS | Q2722 | Operation of Umbilical Hernia-Others |
| 1 | GS | Q2756 | Operation of Inguinal Hernia (Others)-High Ligation And Posterior Repair |
| 1 | GS | Q2757 | Operation of Femoral Hernia |
| 1 | GS | Q2871 | Operation of Internal Bowel Hernia-Reduction |
| 1 | GS | Q2881 | Operation of Periproctal Abscess (Superficial)-Incision And Drainage |
| 1 | GS | Q2883 | Operation of Periproctal Abscess-Deep |
| 1 | GS | QA753 | Operation of Recurrent Inguinal Hernia (With Resection of Intestine)-High Ligation |
| 1 | GS | QA756 | Operation of Recurrent Inguinal Hernia (Others)-High Ligation And Posterior Repair |
| 1 | NS | S4756 | CNS Stereotactic Operation-Biopsy, Aspiration, Excision of Lesion, Hematoma Removal |
| 1 | NS | S0471 | Implantation of Intracranial Neurostimulator Electrodes |
| 1 | NS | S0472 | Implantation of Electrical Stimulator |
| 1 | NS | S0474 | Exchange of Electrical Stimulator |
| 1 | NS | S4741 | Endoscopic Brain Surgery-For Diagnosis |
| 1 | NS | S4722 | Repair of CSF Leakage-Intraspinal |
| 1 | NS | M6599 | Percutaneous Cerebral Angioplasty with Drug |
| 1 | OPH | S5191 | Incision of Orbital Abscess-Invasive |
| 1 | OS | N0233 | Removal of Subcutaneous Benign Tumor |
| 1 | OS | N0317 | Osteotomy-Metacarpal, Metatarsal, Finger, Toe |
| 1 | OS | N0606 | Closed Pinning[Metacarpal,Metatarsal,Finger,Toe] |
| 1 | CS | O1532 | Removal of Chest Wall Foreign Body (Others) |
| 1 | DNT | U4841 | Open Reduction of Mandibular Fracture (Symphysis, Body, Angle of Mandible) |
| 1 | DNT | U4940 | Arthroplasty of TMJ |
| 1 | DNT | UX044 | TMJ Arthrocentesis |
| 1 | ENT | P2113 | Neck Lymphatic Dissection-Unilateral (Modified Radical) |
| 1 | ENT | P2114 | Neck Lymphatic Dissection-Unilateral (Selective) |
| 1 | ENT | P2118 | Neck Lymphatic Dissection-Bilateral |
| 1 | ENT | P2116 | Neck Lymphatic Dissection-Unilateral (Modified Radical) |
| 1 | ENT | P2117 | Neck Lymphatic Dissection-Unilateral (Selective) |
| 1 | ENT | P2119 | Neck Lymphatic Dissection-Bilateral |
| 1 | URO | R3235 | Ureteroenterocutaneostomy-Incontinent |
| 1 | URO | R3236 | Ureteroenterocutaneostomy-Continent |
| 1 | URO | R3600 | Bladder Neck Plasty |
| 1 | URO | R3977 | Holmium Laser Enucleation of The Prostate (HoLEP) |
| 2 | OPH | S5070 | Intravitreal Injection |
| 2 | OS | N0922 | Fasciotomy, Simple |
| 2 | OS | N0923 | Fasciotomy, Complex |
| 2 | OS | N0041 | Escharectomy[9% Under]-Hand, Foot, Finger or Toe |
| 2 | URO | R3515 | Transurethral Bladder Surgery-Coagulation of Bladder Lesion |
| 2 | URO | R3530 | Transurethral Resection of Bladder Neck |
| 2 | URO | R3540 | Transurethral Bladder Surgery-Removal of Blood Clot In Bladder |
| 2 | URO | R3576 | Cystostomy-Percutaneous |
| 2 | URO | R3321 | Nephrostomy-Percutaneous |
| 2 | URO | R3192 | Percutaneous Ureteral Dilatation |
| 2 | CS | Q2333 | Esophagostomy |
| 2 | CS | N0542 | Excision of Others |
| 2 | CS | O1485 | Resection of Chest Wall Tumor, Others (Benign) |
| 2 | CS | Q2352 | Repair of Hiatal Hernia-Abdominal Approach |
| 2 | CS | O0211 | Implantation of Cardioverter Defibrillator-Transvenous-Implantation of Cardioverter Defibrillator |
| 2 | CS | O1530 | Reconstructive Repair of Pectus Excavatum (Ravitch Procedure), Carinatum |
| 2 | CS | O1371 | Revision of Tracheostoma, Simple |
| 2 | CS | O1372 | Revision of Tracheostoma With Local Flap |
| 2 | DNT | U4533 | Surgery of Osteomyelitis of Mandible or Maxilla-Limited Alveolar Bone |
| 2 | DNT | U4534 | Surgery of Osteomyelitis of Mandible or Maxilla-One Side Mandible 1/3 Below |
| 2 | DNT | U4535 | Surgery of Osteomyelitis of Mandible or Maxilla-One Side Mandible 1/3 Over |
| 2 | DNT | U4456 | Intraoral Antiphlogosis-Abscess of Tongue or Mouth of Floor |
| 2 | DNT | U4622 | Oroantral Fistula Closure with Pedicled Flap |
| 2 | ENT | S5711 | Decompression of Facial Nerve-Transauricular Approach |
| 2 | ENT | S5750 | Petrosectomy |
| 2 | ENT | S5800 | Cochlear Implant |
| 2 | ENT | Q2231 | Removal of Parotid Tumor-Benign |
| 2 | ENT | Q2291 | Excision of Oropharyngeal Benign Tumor |
| 2 | GS | Q2613 | Gastrostomy (Invasive)-Transient |
| 2 | GS | Q2614 | Gastrostomy (Invasive)-Permanent |
| 2 | GS | Q2640 | Enterotomy |
| 2 | GS | Q2861 | Appendectomy-Simple |
| 2 | GS | Q2863 | Appendectomy-Removal of Appendical Abscess With Periappendical Abscess Drainage |
| 2 | GS | Q2862 | Appendectomy-Perforated |
| 2 | GS | Q2801 | Repair of Enteric Fistula-Loop |
| 2 | GS | Q2802 | Repair of Enteric Fistula-End |
| 2 | GS | Q2803 | Repair of Enteric Fistula-Double Barrel |
| 2 | GS | Q2804 | Repair of Enteric Fistula-Reversal of Hartmann Procedure |
| 2 | GS | Q7390 | Cholecystostomy, Cholecystotomy |
| 2 | GS | O2011 | External AV Shunt For Hemodialysis |
| 2 | GS | P4571 | Adrenalectomy-Unilateral |
| 2 | GS | Q2551 | Vagotomy (Truncal Vagotomy)-With Gastrojejunostomy or Pyloroplasty |
| 2 | GS | Q2645 | Polypectomy of Small Bowel or Colon |
| 2 | GS | Q2561 | Pyloroplasty (Fredet-Ramstedt Pyloromyotomy) |
| 2 | GS | Q2562 | Pyloroplasty (Others) |
| 2 | GS | O2081 | Fistula Formation-Autologus Vein For Hemodialysis |
| 2 | GS | O2082 | Fistula Formation-Artificial Vein For Hemodialysis |
| 2 | GS | O2083 | Repair of Arterio-Venous Fistula For Hemodialysis |
| 2 | GY | R4421 | Extirpation of Adnexal Tumor-Benign |
| 2 | GY | R4148 | Hysterectomy (without Lymphadenectomy)-Abdominal approach-complex |
| 2 | GY | R0409 | Colporrhaphy-Anterior Colporrhaphy |
| 2 | GY | R4070 | Extirpation of Vaginal Tumor-Benign |
| 2 | GY | R4112 | Colpopexy (Surgical)-Vaginal Approach |
| 2 | GY | R4157 | Pelvic And Para-Aortic Lymphadenectomy |
| 2 | GY | R4130 | Subtotal Hysterectomy |
| 2 | GY | R4143 | Hysterectomy (With Lymphadenectomy)-Simple |
| 2 | GY | R4202 | Vaginal Total Hysterectomy |
| 2 | GY | R4203 | Vaginal Total Hysterectomy With A And P Repair |
| 2 | CS | M6521 | Percutaneous Atrial Septostomy-Balloon |
| 2 | CS | OZ751 | Percutaneous Closure of Interatrial Septal Defect |
| 2 | CS | M6511 | Percutaneous Left Atrial Appendage Occlusion |
| 2 | NS | N1497 | Laminectomy, Cervical Spine |
| 2 | NS | N1498 | Laminectomy, Thoracic Spine |
| 2 | NS | N1499 | Laminectomy, Lumbar Spine |
| 2 | NS | N2491 | Cervical Spine Laminoplasty |
| 2 | NS | N2492 | Cervical Spine Laminoplasty |
| 2 | NS | N2497 | Laminectomy, Cervical Spine |
| 2 | NS | N2498 | Laminectomy, Thoracic Spine |
| 2 | NS | N2499 | Laminectomy, Lumbar Spine |
| 2 | NS | N0455 | Surgical removal of the ossification of spinal ligament (OLF removal-posterior approach) |
| 2 | OPH | S4900 | Enucleation |
| 2 | OPH | S5220 | Enucleation and Tissue Implantation |
| 2 | OS | N0685 | Arthrotomy For Acute Septic Joint[Elbow,Wrist,Ankle] |
| 2 | OS | N0688 | Arthrotomy For Acute Septic Joint[Elbow,Wrist,Ankle] |
| 2 | OS | N0686 | Arthrotomy For Acute Septic Joint[Finger,Toe] |
| 2 | OS | N0689 | Arthrotomy For Acute Septic Joint[Finger,Toe] |
| 2 | OS | N2072 | Replacement Arthroplasty-Total[Knee] |
| 2 | OS | N2077 | Replacement Arthroplasty-Total[Knee] |
| 2 | OS | N2712 | Replacement Arthroplasty-Hemiarhtroplasty[Knee] |
| 2 | OS | N3715 | Revision of Total Arthroplasty[Ankle] |
| 2 | OS | N0311 | Ostectomy |
| 2 | OS | N0312 | Bone Graft |
| 2 | OS | N0604 | Open Reduction of Fractured Extremity[Clavicle,Patella,Carpal Bone,Tarsal Bone] |
| 2 | OS | N0605 | Open Reduction of Fractured Extremity[Metacarpal,Metatarsal,Finger,Toe] |
| 2 | OS | N0614 | Open Reduction of Fractured Extremity[Clavicle,Patella,Carpal Bone,Tarsal Bone] |
| 2 | OS | N0622 | Repair of Nonunion or Malunion[Humerus,Forearm,Tibia] |
| 2 | OS | N0703 | Excision of Joint[Including Synovectomy]-Elbow,Wrist,Ankle |
| 2 | OS | N0704 | Excision of Joint[Including Synovectomy]-Finger,Toe |
| 2 | OS | N0708 | Excision of Joint[Including Synovectomy]-Elbow,Wrist,Ankle |
| 2 | OS | N0710 | Excision of Joint [Including Synovectomy]-Knee |
| 2 | OS | N0723 | Resection Arthroplasty[Finger,Toe] |
| 2 | OS | N0733 | Arthrodesis[Elbow,Wrist,Ankle] |
| 2 | OS | N0734 | Arthrodesis[Finger,Toe] |
| 2 | OS | N0911 | Subcutaneous Tenotomy |
| 2 | OS | N0931 | Reconstruction of Tendon And Ligament, Simple |
| 2 | OS | N0932 | Reconstruction of Tendon And Ligament, Complex |
| 2 | OS | N0936 | Acromioplasty And Repair of Ruptured Shoulder Rotator Cuff (Primary Repair) |
| 2 | OS | N0937 | Acromioplasty And Repair of Ruptured Shoulder Rotator Cuff (With Myoplasty And Tendoplasty) |
| 2 | OS | N0941 | Tenolysis |
| 2 | OS | N0981 | External Fixation[Pelvis,Femur] |
| 2 | OS | N0982 | External Fixation[Humerus,Tibia] |
| 2 | OS | N0986 | External Fixation[Humerus,Tibia] |
| 2 | OS | N1584 | Vascularized Osteocutaneous Graft |
| 2 | OS | N1601 | Open Reduction of Fractured Extremity-Forearm (Radius) |
| 2 | OS | N1604 | Open Reduction of Fractured Extremity-Crus (Tibia) |
| 2 | OS | N1611 | Open Reduction of Fractured Extremity-Forearm (Radius) |
| 2 | OS | N1612 | Open Reduction of Fractured Extremity-Forearm (Ulnar) |
| 2 | OS | N1616 | Open Reduction of Fractured Extremity-Crus (Tibia and Fibula) |
| 2 | OS | S0165 | Muscle Flap-Others |
| 2 | OS | S0166 | Musculocutaneous Flap or Myocutaneous Flap-Others |
| 2 | OS | S0168 | Fasciocutaneous Flap-Others |
| 2 | OS | N2075 | Replacement Arthroplasty-Total[Ankle] |
| 2 | OS | N2078 | Replacement Arthroplasty-Total[Elbow] |
| 2 | OS | N0565 | Disarticulation of Extremities[Elbow, Wrist, Ankle] |
| 2 | OS | N0566 | Disarticulation of Extremities[Finger, Toe] |
| 2 | OS | N0582 | Revision of Amputated Stump (Need Osteoplasty)-Finger, Toe |
| 2 | OS | N0584 | Revision of Amputated Stump (Need Plastic Operation On Soft Tissue)-Finger, Toe |
| 2 | OS | N0752 | Open Reduction of Dislocation[Shoulder] |
| 2 | OS | N0282 | Curettage or Excision of Benign Bone Tumor[Humerus, Forearm Bone, Clavicle] |
| 2 | OS | N0581 | Revision of Amputated Stump (Need Osteoplasty)-Thigh, Lower Leg, Upper Arm, Forearm |
| 2 | OS | N0583 | Revision of Amputated Stump (Need Plastic Operation On Soft Tissue)-Thigh, Lower Leg, Upper Arm, Forearm |
| 2 | OS | N0844 | Drainage of Intramuscular Abscess (Others) |
| 2 | PS | N0952 | Operation of Fractured Lower Jaw (Open Reduction)-Symphysis, Body, Angle of Mandible |
| 2 | PS | N0152 | Wide Excision And Lymph Node Dissection |
| 2 | URO | R3542 | Transurethral Bladder Surgery-Tumor (Complex) |
| 2 | URO | R3543 | Transurethral Bladder Surgery-Tumor (Highly Complex) |
| 2 | URO | R3290 | Partial Nephrectomy |
| 2 | URO | R3950 | Prostatectomy |
| 2 | URO | R3151 | Ureteroneocystostomy-Simple |
| 2 | URO | R3154 | Ureteroneocystostomy-Using Bladder Flap |
| 2 | URO | R3180 | End-To-End Ureteroureterostomy |
| 2 | URO | R3231 | Uretero-Intestino-Vesical Anastomosis |
| 2 | URO | R3421 | Operative Ureterolithotomy-Upper |
| 2 | URO | R3620 | Repair of Cystocele |
| 2 | URO | R3683 | Repair of Urethral Stricture-Transpubic |
| 2 | URO | R3720 | External Urethral Meatotomy |
| 2 | URO | R3755 | Urethrectomy-Male |
| 2 | URO | R3756 | Urethrectomy-Female |
| 2 | URO | R3770 | Removal of Urethral Caruncle |
| 2 | URO | R3896 | Operation of Vas Deferens-Vasectomy Or Ligation |
| 2 | URO | R3400 | Pyeloplasty, Ureteroplasty |
| 2 | URO | R3433 | Ureterectomy-Removal of Remnant Ureter |
| 2 | URO | R3461 | Cystotomy-Stone Or Foreign Body |
| 2 | URO | R3791 | Penectomy-Partial |
| 2 | URO | R3792 | Penectomy-Total |
| 2 | URO | R3851 | Orchiectomy-Total |
| 2 | URO | R3853 | Orchiectomy-Partial |
| 2 | URO | R3902 | Excision of Scrotum-Partial |
| 2 | GY | R4427 | Extirpation of Adnexal Tumor (Malignant)-Simple |
| 2 | NS | N1466 | Arthrodesis of Spine-Lumbar Spine-Anterior Technique |
| 2 | NS | N1460 | Posterior Lumbar Interbody Fusion |
| 2 | NS | N1469 | Arthrodesis of Spine-Lumbar Spine-Posterior Technique |
| 2 | NS | N2468 | Arthrodesis of Spine-Cervical Spine-Posterior Technique[C1-2 Fixation] |
| 2 | NS | N0466 | Arthrodesis of Spine-Lumbar Spine-Anterior Technique |
| 2 | NS | N0469 | Arthrodesis of Spine-Lumbar Spine-Posterior Technique |
| 2 | NS | N2463 | Arthrodesis of Spine-Cervical Spine-Anterior Technique[Others] |
| 2 | NS | N2466 | Arthrodesis of Spine-Thoracic Spine-Anterior Technique[Others] |
| 2 | NS | N0468 | Arthrodesis of Spine-Thoracic Spine-Posterior Technique |
| 2 | NS | N2469 | Arthrodesis of Spine-Cervical Spine-Posterior Technique[Others] |
| 2 | NS | N2470 | Posterior Lumbar Interbody Fusion |
| 2 | OS | N0602 | Open Reduction of Fractured Extremity[Humerus,Scapula] |
| 2 | OS | N0612 | Open Reduction of Fractured Extremity[Humerus,Scapula] |
| 2 | OS | N3712 | Revision of Total Arthroplasty[Knee] |
| 2 | OS | N3722 | Revision of Total Arthroplasty[Knee] |
| 2 | OS | N4712 | Revision of hemiarthroplasty[Knee] |
| 2 | OS | N4717 | Revision of hemiarthroplasty[Knee] |
| 2 | URO | R3975 | Transurethral Resection of Prostate |
| 2 | CS | Q2341 | Suture of Esophagus-Cervical Approach |
| 2 | CS | Q2342 | Suture of Esophagus-Thoracic Approach |
| 2 | DNT | U4861 | Partial Mandibulectomy |
| 2 | ENT | P2112 | Neck Lymphatic Dissection-Unilateral (Radical) |
| 2 | ENT | P2115 | Neck Lymphatic Dissection-Unilateral (Radical) |
| 2 | ENT | Q2201 | Removal of Tumor In Oral Cavity-Benign |
| 2 | ENT | Q2181 | Operation for Malignant Tongue Tumor (Less Than One-Half Tongue) |
| 2 | GS | Q7566 | Pancreatectomy-Wedge Resection |
| 2 | GY | R4423 | Extirpation of Adnexal Tumor (Malignant)-Simple |
| 2 | NS | S4625 | Intracranial Foreign Body Removal |
| 2 | NS | M6636 | Percutaneous Thrombus Removal-Mechanical thrombectomy (Intracranial vessel) |
| 2 | NS | M6637 | Percutaneous Thrombus Removal-Mechanical thrombectomy (Extracranial cervical vessel) |
| 2 | NS | M6639 | Percutaneous Thrombus Removal-Mechanical thrombectomy (Others) |
| 2 | NS | S4709 | Excision of Extradural Tumor Or Lesion-Lumbar Spine-Without Pedicle And Vertebral Body |
| 2 | NS | S6696 | Excision of Intradural Tumor Or Lesion-Lumbar Spine |
| 2 | OS | N2071 | Replacement Arthroplasty-Total[Shoulder] |
| 2 | OS | N2076 | Replacement Arthroplasty-Total[Shoulder] |
| 2 | OS | N3716 | Revision of Total Arthroplasty[Shoulder] |
| 2 | OS | N0701 | Excision of Joint[Including Synovectomy]-Hip |
| 2 | OS | N0751 | Open Reduction of Dislocation[Hip] |
| 2 | PS | N0391 | Partial Maxillectomy |
| 2 | PS | N0404 | Partial Maxillectomy, Malignant Tumor |
| 2 | PS | N0421 | Partial Mandibulectomy |
| 2 | PS | N0436 | Resection of Malignant Tumor (Partial Mandibulectomy) |
| 2 | GS | P4561 | Radical Operation of Malignant Thyroid Tumor |
| 2 | GY | R4154 | Radical Hysterectomy With Bilateral Pelvic Lymphadenectomy (With Para-Aortic Lymph Node Biopsy) |
| 2 | GY | R4155 | Radical Hysterectomy With Bilateral Pelvic Lymphadenectomy (Without Para-Aortic Lymph Node Biopsy) |
| 2 | GY | R4160 | Pelvic Adhesiolysis |
| 2 | GY | R4424 | Extirpation of Adnexal Tumor (Malignant)-Radical |
| 2 | GY | R4428 | Extirpation of Adnexal Tumor (Malignant)-Radical |
| 2 | NS | N0342 | Cranioplasty With Dura Graft |
| 2 | NS | N0347 | Cranioplasty-Complex |
| 2 | URO | R3910 | Radical Hydrocelectomy |
| 2 | GS | OA633 | Angioplasty (End-to-End Anastomosis)-Others |
| 2 | GS | OA638 | Angioplasty (With Patch Graft)-Others,Artificial Vessel |
| 2 | GS | OA639 | Angioplasty (With Patch Graft)-Others,Autologous Vessel |
| 2 | GS | Q7703 | Colonoscopic Operation Of Colonic Tumor-Mucosal Resection And Submucosal Resection |
| 2 | OS | N0575 | Amputation of Extremities[Finger,Toe] |
| 2 | PS | N0151 | Wide Excision of Skin Malignant Tumor |
| 2 | GS | Q7430 | Excision of Ampulla of Vater & Replantation of Bile & Pancreatic Duct |
| 2 | OS | N2070 | Total Arthroplasty[Hip] |
| 2 | OS | N0711 | Total Arthroplasty[Hip] |
| 2 | OS | N2710 | Hemiarthroplasty[Hip] |
| 2 | OS | N0715 | Hemiarthroplasty[Hip] |
| 2 | URO | R3960 | Total Prostatoseminal Vesiculectomy |
| 2 | OS | N1715 | Revision of Hemiarthroplasty[Hip] |
| 2 | OS | N1725 | Revision of Hemiarthroplasty[Hip] |
| 2 | OS | N1711 | Revision of Total Arthroplasty[Hip] |
| 2 | OS | N1721 | Revision of Total Arthroplasty[Hip] |
| 2 | OS | N3710 | Revision of Total Arthroplasty[Hip] |
| 2 | OS | N3720 | Revision of Total Arthroplasty[Hip] |
| 2 | OS | N4710 | Revision of Hemiarthroplasty[Hip] |
| 2 | OS | N4720 | Revision of Hemiarthroplasty[Hip] |
| 2 | OS | N0684 | Arthrotomy For Acute Septic Joint[Shoulder,Knee] |
| 2 | ENT | Q2232 | Removal of Parotid Tumor-Malignant |
| 2 | GS | Q2775 | Primary Repair of Mesentery |
| 3 | GS | M6690 | Percutaneous Cholecystostomy |
| 3 | GS | M6730 | Percutaneous Gastrostomy |
| 3 | GS | M6741 | Percutaneous Abscess Drainage |
| 3 | GS | M6750 | Percutaneous Drainage Catheter Exchange |
| 3 | GS | Q2612 | Gastrostomy (Percutaneous) |
| 3 | ENT | O1221 | Resection of Laryngeal Benign Tumor-Under Endoscopy-Under Suspension Laryngoscopy |
| 3 | GS | M6670 | Percutaneous Transhepatic Biliary Drainage |
| 3 | GS | M6681 | Percutaneous Transhepatic Biliary Drainage With Ballooning Catheter |
| 3 | GS | M6682 | Percutaneous Transhepatic Biliary Drainage With Stent |
| 3 | CS | O1510 | Closed Thoracostomy |
| 3 | CS | O2005 | Transcutaneous Cardiac Pacing |
| 3 | CS | O1571 | Closure of Sternotomy Separation |
| 3 | ENT | P2102 | Excision of Cervical Lymph Node-Superficial |
| 3 | ENT | P2103 | Excision of Cervical Lymph Node-Deep |
| 3 | GS | Q2810 | Adhesiolysis |
| 3 | GS | Q2680 | Intestinal Anastomosis |
| 3 | GS | Q2753 | Operation of Inguinal Hernia (With Resection of Intestine)-High Ligation |
| 3 | GS | Q2731 | Operation of Incisional Hernia-With Resection of Intestine |
| 3 | GS | Q2732 | Operation of Incisional Hernia-Others |
| 3 | GS | Q2754 | Operation of Inguinal Hernia (With Resection of Intestine)-High Ligation And Posterior Repair |
| 3 | GS | Q2791 | Enterostomy-Tube |
| 3 | GS | Q2792 | Enterostomy-Loop |
| 3 | GS | Q2793 | Enterostomy-End |
| 3 | GS | Q2794 | Enterostomy-Double Barrel |
| 3 | GS | Q2796 | Enterostomy-Revision of Ileostomy or Colostomy (Revision of loop ileostomy) |
| 3 | GS | Q2797 | Enterostomy-Revision of Ileostomy or Colostomy (Revision of loop colostomy) |
| 3 | GS | Q2798 | Enterostomy-Revision of Ileostomy or Colostomy (Revision of End ileostomy or End colostomy) |
| 3 | GS | Q7380 | Cholecystectomy |
| 3 | GS | O2073 | Vessel Ligation Others |
| 3 | GS | O2074 | Vessel Ligation Others |
| 3 | OS | N0232 | Removal of Malignant Tumor |
| 3 | URO | R3271 | Nephrectomy-Simple |
| 3 | URO | R3571 | Cystostomy-Operative |
| 3 | URO | R3470 | Partial Cystectomy |
| 3 | URO | R3920 | Incision of Scrotal Abscess |
| 3 | CS | O1610 | Repair of Diaphragmatic Hernia |
| 3 | CS | N0550 | Radical Curettage of Thoracic Cold Abscess |
| 3 | CS | O1562 | Sternum Resection And Reconstruction |
| 3 | CS | O1600 | Repair of Diaphragm |
| 3 | CS | Q2424 | Esophageal Diverticulectomy-Thoracic Approach |
| 3 | CS | O1520 | Open Thoracostomy |
| 3 | ENT | S5592 | Excision of External Auditory Canal Tumor-Malignant |
| 3 | GS | Q2710 | Intestinal Plication |
| 3 | GS | Q2510 | Gastrotomy |
| 3 | NS | M1661 | Embolization-Cerebral/Aneurysm/Assisted |
| 3 | NS | M1662 | Embolization-Cerebral/Aneurysm/Others |
| 3 | NS | M6601 | Percutaneous Intravascular Installation of Metallic Stent-Cerebral |
| 3 | NS | M6602 | Percutaneous Intravascular Installation of Metallic Stent-Carotid |
| 3 | OS | N0574 | Amputation of Extremities[Hand,Foot] |
| 3 | OS | N0591 | Open Reduction of Fracture and Dislocation of Spine or Pelvis-Spine |
| 3 | OS | N0601 | Open Reduction of Fractured Extremity[Femur] |
| 3 | OS | N0611 | Open Reduction of Fractured Extremity[Femur] |
| 3 | NS | M1665 | Embolization-Cerebral/Arteriovenous Malformation/Dural Arteriovenous Fistula/Transvenous |
| 3 | NS | M1666 | Embolization-Cerebral/Arteriovenous Malformation/Caroticocavernous Fistula |
| 3 | NS | M1675 | Embolization-Tumor/Spinal |
| 3 | OS | N0022 | Operation of Osteomyelitis or Bone Abscess[Drilling, Fenestration Etc]-Humerus,Forearm Bone,Clavicle |
| 3 | OS | N0023 | Operation of Osteomyelitis or Bone Abscess[Drilling, Fenestration, Saucerization Etc]-Others |
| 3 | OS | N0025 | Operation of Osteomyelitis or Bone Abscess[Drilling, Fenestration Etc]-Humerus,Forearm Bone,Clavicle |
| 3 | OS | N0026 | Operation of Osteomyelitis or Bone Abscess[Drilling, Fenestration, Saucerization Etc]-Others |
| 3 | OS | N0021 | Operation of Osteomyelitis or Bone Abscess[Drilling, Fenestration Etc]-Pelvis,Femur,Tibia |
| 3 | URO | R3241 | Augmentation Enterocystoplasty |
| 3 | URO | R3482 | Total Cystectomy-Others |
| 3 | URO | R3273 | Nephrectomy-Radical |
| 3 | ENT | Q2182 | Operation for Malignant Tongue Tumor-Hemiglossectomy |
| 3 | ENT | Q2203 | Removal of Tumor In Oral Cavity-Malignant |
| 3 | CS | O1401 | Wedge Resection of Lung, Single |
| 3 | GS | O0161 | Vascular Bypass Operation (Femoral-Femoral, Clavicle-Clavicle Or Axilla-Axilla),Autologous Vessel |
| 3 | GS | O0162 | Vascular Bypass Operation (Femoral-Femoral, Clavicle-Clavicle Or Axilla-Axilla),Artificial Vessel |
| 3 | GS | O0163 | Vascular Bypass Operation (Femoral-Popliteal[Above Knee Joint]),Autologous Vessel |
| 3 | GS | O0164 | Vascular Bypass Operation (Femoral-Popliteal[Knee Joint Upper]),Artificial Vessel |
| 3 | GS | O0166 | Vascular Bypass Operation (Femoral-Popliteal[Below Knee Joint]),Artificial Vessel |
| 3 | GS | Q2490 | Incision And Drainage of Subphrenic Abscess |
| 3 | GS | P2094 | Splenorrhaphy |
| 3 | CS | M6620 | Percutaneous Intravascular Installation of Metallic Stent-Percutaneous Intravascular Atherectomy |
| 3 | CS | M6597 | Percutaneous Transluminal Angioplasty-Others |
| 3 | CS | M6613 | Percutaneous Intravascular Installation of Stent Graft-Others |
| 3 | NS | S4705 | Excision of Extradural Tumor Or Lesion-Cervical Spine-Without Pedicle And Vertebral Body |
| 3 | NS | S6692 | Excision of Intradural Tumor Or Lesion-Cervical Spine |
| 3 | NS | S4706 | Excision of Extradural Tumor Or Lesion-Thoracic Spine-Involving Pedicle And/Or Vertebral Body |
| 3 | OS | N0680 | Arthrotomy For Acute Septic Joint[Hip] |
| 3 | OS | N0681 | Arthrotomy For Acute Septic Joint[Hip] |
| 4 | URO | R3432 | Ureterectomy-Total Nephroureterectomy |
| 4 | GS | Q2691 | Operation for Intestinal Obstruction-Including Resection of Intestine |
| 4 | GS | Q2692 | Operation for Intestinal Obstruction-Entero-Enterostomy |
| 4 | GS | Q2693 | Operation for Intestinal Obstruction-Adhesiolysis |
| 4 | GS | Q7310 | Choledochotomy And Choledocholithotomy |
| 4 | CS | M6632 | Percutaneous Thrombus Removal-Thrombolysis-Others |
| 4 | URO | R3251 | Intestinal Substitute of Bladder |
| 4 | URO | R3272 | Donor Nephrectomy |
| 4 | URO | R3550 | Repair of Bladder Rupture |
| 4 | CS | O1605 | Excision of Diaphragmatic Tumor And Reconstruction |
| 4 | CS | O1645 | Vascular Bypass Operation (Artery-Others),Autologous Vessel |
| 4 | CS | O1646 | Vascular Bypass Operation (Artery-Others),Artificial Vessel |
| 4 | CS | O1581 | Mediastinostomy, Collor's |
| 4 | CS | O2035 | Resection of Aneurysm-Others |
| 4 | CS | O2037 | Resection of Aneurysm-Iliac Artery (Bilateral) |
| 4 | CS | O2038 | Resection of Aneurysm-Iliac Artery (Unilateral) |
| 4 | CS | O1486 | Resection of Chest Wall Tumor, Others (Malignant) |
| 4 | CS | O1484 | Resection of Chest Wall Tumor With Reconstruction of Chest Wall (Malignant) |
| 4 | DNT | U4465 | Extraoral Antiphlogosis-Deep Layer |
| 4 | DNT | U4812 | Total Maxillectomy, Malignant Tumor |
| 4 | ENT | O1251 | Total Laryngectomy And Hypopharyngectomy |
| 4 | ENT | O1252 | Total Laryngectomy And Partial Hypopharyngectomy |
| 4 | ENT | Q2251 | Incision of Retropharyngeal Abscess-Intraoral |
| 4 | ENT | Q2252 | Incision of Retropharyngeal Abscess-Transcervical |
| 4 | ENT | O0962 | Extirpation of Malignant Tumor of Nasal or Paranasal Sinuses (Radical Maxillectomy) |
| 4 | GS | Q2762 | Excision of Mesenteric Tumor-Others |
| 4 | GS | Q2502 | Excision of Retroperitoneal Tumor-Malignant or Pheochromocytoma |
| 4 | GS | P2091 | Splenectomy-Total |
| 4 | GS | Q7221 | Hepatectomy-Wedge Resection |
| 4 | GS | O2064 | Transluminal Atherectomy-Abdominal Artery or Iliac Artery |
| 4 | GS | O2067 | Transluminal Atherectomy-Abdominal Artery or Iliac Artery |
| 4 | GS | Q0257 | Subtotal Gastrectomy (Wedge Resection) |
| 4 | GS | O2072 | Vessel Ligation Following Laparotomy |
| 4 | GS | Q7563 | Subtotal Pancreatectomy |
| 4 | GS | Q7565 | Distal Pancreatectomy |
| 4 | GS | OA632 | Angioplasty (End-to-End Anastomosis)-By Laparotomy |
| 4 | GS | OA636 | Angioplasty (With Patch Graft)-By Laparotomy,Artificial Vessel |
| 4 | GS | OA637 | Angioplasty (With Patch Graft)-By Laparotomy,Autologous Vessel |
| 4 | GS | Q2673 | Colectomy-Segmental Resection |
| 4 | GS | QA673 | Colectomy-Segmental Resection |
| 4 | GS | QA679 | Colectomy With Proximal Colostomy And Distal Stump |
| 4 | GS | QA921 | Rectal And Sigmoid Resection-Anterior Resection |
| 4 | GS | QA922 | Rectal And Sigmoid Resection-Low Anterior Resection |
| 4 | GS | QA923 | Rectal And Sigmoid Resection[A-P Resection (Mile's Operation) or A-S Resection] |
| 4 | GS | Q2892 | Resection of Rectal Tumor-Abdominal Approach |
| 4 | GS | Q2921 | Rectal And Sigmoid Resection-Anterior Resection |
| 4 | GS | Q2922 | Rectal And Sigmoid Resection-Low Anterior Resection |
| 4 | GS | Q2923 | Rectal And Sigmoid Resection[A-P Resection (Mile's Operation) or A-S Resection] |
| 4 | GS | QA928 | Rectal and Sigmoid Resection-Ultra-Low anterior resection |
| 4 | GS | Q2481 | Peritoneal Lavage |
| 4 | GS | Q2540 | Simple Closure of Perforated Stomach or Duodenum |
| 4 | GY | R4156 | Pelvic Exenteration |
| 4 | CS | M6605 | Percutaneous Intravascular Installation of Metallic Stent-Others |
| 4 | GS | O2065 | Transluminal Atherectomy-Others |
| 4 | GS | O2068 | Transluminal Atherectomy-Others |
| 4 | NS | S4711 | Shunt Operation Or Bypass Operation-Subdural/Subarachnoid-Other |
| 4 | NS | S4712 | Shunt Operation Or Bypass Operation-Ventriculo-Other |
| 4 | NS | S4801 | Operation of Skull Base-Anterior Cranial Fossa |
| 4 | NS | N0321 | Burr Hole or Trephination For Exploration |
| 4 | NS | N0322 | Burr Hole or Trephination For Drainage And/Or Evacuation of Cyst,Hematoma or Abscess (Sub or Epidural) |
| 4 | NS | N0323 | Burr Hole or Trephination For Drainage And/Or Evacuation of Cyst,Hematoma or Abscess (Intracerebral) |
| 4 | NS | N0324 | Burr Hole or Trephination For Others |
| 4 | URO | R3481 | Total Cystectomy-Radical |
| 4 | NS | N0451 | Vertebral Corpectomy (Cervical Spine) |
| 4 | NS | N0453 | Vertebral Corpectomy (Lumbar Spine) |
| 4 | NS | M6644 | Embolization-Others |
| 4 | NS | S4661 | Intracerebral Vascular Anastomosis-Direct |
| 4 | NS | N0452 | Vertebral Corpectomy (Thoracic Spine) |
| 5 | GS | Q2440 | Diagnostic Exploratory Laparotomy |
| 5 | CS | O1502 | Irrigation of Empyema Cavity |
| 5 | CS | O1981 | Resection of Atrial Myxoma |
| 5 | CS | O1982 | Resection of Cardiac Tumor-Others |
| 5 | CS | O1596 | Mediasternal Lymph Node Dissection |
| 5 | CS | O1597 | Mediasternal Lymph Node Dissection |
| 5 | CS | O1591 | Excision of Mediastinal Benign Tumor |
| 5 | CS | O1410 | Segmentectomy of Lung |
| 5 | CS | O1586 | Mediastinostomy By Thoracotomy |
| 5 | CS | O1336 | Removal of Tracheal or Bronchial Foreign Body By Thoracotomy |
| 5 | CS | O2004 | Implantation of Internal Pulse Generator By Thoracotomy |
| 5 | CS | O1321 | Tracheal or Bronchial Repair, Cervical Approach |
| 5 | CS | O2006 | Operation of Arrhythmia-Supraventricular Arrhythmia |
| 5 | CS | M6542 | Conventional Radiofrequency Ablation of Atrial fibrillation |
| 5 | CS | M6545 | Conventional Radiofrequency Ablation of Atrial fibrillation with Septal Puncture |
| 5 | CS | O1440 | Repair of Lung |
| 5 | ENT | O1224 | Resection of Laryngeal Malignant Tumor (Cordectomy) |
| 5 | ENT | O1225 | Partial Laryngectomy (Vertical, Supraglottic) |
| 5 | ENT | O1227 | Resection of Laryngeal Malignant Tumor (Total Laryngectomy) |
| 5 | ENT | Q2292 | Operation for Pharyngeal Malignant Tumor (Oropharyngeal)-Simple Resection |
| 5 | ENT | Q2293 | Operation for Pharyngeal Malignant Tumor (Oropharyngeal)-Composite Resection |
| 5 | ENT | Q2294 | Operation for Pharyngeal Malignant Tumor-Partial Hypopharyngectomy |
| 5 | ENT | O1300 | Invasive Tracheostomy |
| 5 | ENT | O0226 | Transluminal Atherectomy-Carotid Artery (Simple) |
| 5 | ENT | O0227 | Transluminal Atherectomy-Carotid Artery (Complex) |
| 5 | ENT | O2066 | Transluminal Atherectomy-Carotid Artery |
| 5 | GS | Q7410 | Radical Cholecystectomy of GB Cancer |
| 5 | GS | Q0251 | Subtotal Gastrectomy (Partial) |
| 5 | GS | Q0252 | Subtotal Gastrectomy (Distal) |
| 5 | GS | Q0253 | Subtotal Gastrectomy (Distal) |
| 5 | GS | Q0258 | Subtotal Gastrectomy (Proximal Resection) |
| 5 | GS | Q0259 | Subtotal Gastrectomy |
| 5 | GS | Q2598 | Subtotal Gastrectomy (Proximal Resection) |
| 5 | GS | Q2594 | Subtotal Gastrectomy (Partial) |
| 5 | GS | O2045 | Inferior Vena Cava Filter Placement |
| 5 | GS | Q2671 | Right or Left Hemicolectomy |
| 5 | GS | Q1261 | Colectomy-Subtotal |
| 5 | GS | Q1262 | Colectomy-Subtotal |
| 5 | GS | QA671 | Right or Left Hemicolectomy |
| 5 | GS | Q7222 | Hepatectomy-Segmentectomy |
| 5 | GS | Q7225 | Hepatectomy-Bisegmentectomy |
| 5 | GS | Q2650 | Resection of Small Intestine |
| 5 | GS | Q2651 | Resection of Small Intestine |
| 5 | GS | Q7561 | Pancreatectomy-Total |
| 5 | GS | Q7592 | Pancreaticoenterostomy (End-to-End Anastomosis) |
| 5 | GS | Q7351 | Choledochoduodenostomy |
| 5 | GS | Q7352 | Choledochojejunostomy[Roux-en-Y] |
| 5 | GS | Q2572 | Gastrojejunostomy |
| 5 | GS | Q2573 | Gastrojejunostomy[Roux-En-Y] |
| 5 | GS | Q2771 | Repair of Bowel And Mesenteric Injury-With Resection of Intestine |
| 5 | GS | Q2773 | Serosal Repair or Primary Repair of Perforated Intestine |
| 5 | GS | Q2601 | Esophagojejunostomy |
| 5 | CS | M6595 | Percutaneous Transluminal Angioplasty-Aortic |
| 5 | CS | M6603 | Percutaneous Intravascular Installation of Metallic Stent-Aortic |
| 5 | CS | M6611 | Percutaneous Intravascular Installation of Stent Graft-Aortic |
| 5 | CS | M6612 | Percutaneous Intravascular Installation of Stent Graft-Aortic And Iliac |
| 5 | CS | M6551 | Percutaneous Transluminal Coronary Angioplasty-Single Vessel |
| 5 | CS | M6561 | Percutaneous Transcatheter Placement of Intracoronary Stent-Single Vessel |
| 5 | NS | S4636 | Craniotomy for Excision of Brain Tumor-Infratentorial-Simple |
| 5 | NS | S4641 | Cerebral Aneurysm-Simple |
| 5 | NS | S4642 | Cerebral Aneurysm-Complex |
| 5 | OS | N0562 | Disarticulation of Extremities[Hip] |
| 5 | OS | N0573 | Amputation of Extremities[Upper Arm,Forearm,Lower Leg] |
| 6 | CS | O1360 | Exploratory Thoracotomy |
| 6 | GS | Q7342 | Radical Resection of Bile Duct-Malignant |
| 6 | CS | O1592 | Excision of Mediastinal Malignant Tumor |
| 6 | CS | O1841 | Repair of Arteriovenous Malformation-By Thoracotomy |
| 6 | CS | O2071 | Vessel Ligation Following Thoracotomy |
| 6 | CS | OA631 | Angioplasty (End-to-End Anastomosis)-By Thoracotomy |
| 6 | CS | OA634 | Angioplasty (With Patch Graft)-By Thoracotomy,Artificial Vessel |
| 6 | CS | OA635 | Angioplasty (With Patch Graft)-By Thoracotomy,Autologous Vessel |
| 6 | CS | O1421 | Single Lobectomy of Lung |
| 6 | CS | O1422 | Bilobectomy of Lung |
| 6 | CS | O1660 | Repair of Cardiac Wound |
| 6 | CS | O1480 | Pleurodesis |
| 6 | CS | O1460 | Apicolysis, Pleurolysis |
| 6 | CS | O1932 | Creation of Pericardial Window-by Thoracotomy |
| 6 | CS | O1935 | Creation of Pericardial Window-by Microscopy |
| 6 | CS | O1931 | Pericardiostomy |
| 6 | ENT | O2055 | Thrombectomy (Artery),Neck |
| 6 | GS | Q2925 | Total Coloprotectomy (With Ileostomy) |
| 6 | GS | Q2533 | Total Gastrectomy-Abdominal Approach |
| 6 | GS | Q2536 | Total Gastrectomy-Abdominal Approach |
| 6 | GS | Q7223 | Hepatectomy-Lobectomy |
| 6 | GS | O0281 | Removal of Infected Graft-Others |
| 6 | GS | Q7224 | Hepatectomy-Trisegmentectomy |
| 6 | GS | QA925 | Total Coloprotectomy (With Ileostomy) |
| 6 | GS | Q2672 | Colectomy-Total |
| 6 | GS | QA672 | Colectomy-Total |
| 6 | GS | Q2679 | Colectomy With Proximal Colostomy And Distal Stump |
| 6 | GS | O2054 | Thrombectomy (Artery),Abdomen |
| 6 | GS | O0218 | Thrombectomy (Deep Vein),Lower Extremity |
| 6 | GS | O2058 | Thrombectomy (Deep Vein),Abdomen |
| 6 | GS | Q7572 | Pancreaticoduodenectomy-Pylorus-Preserving Operation |
| 6 | GS | Q7571 | Pancreaticoduodenoctomy-Whipple'S Operation |
| 6 | CS | M6650 | Percutaneous Installation of Inferior Vena Cava Filter |
| 6 | CS | M6531 | Percutaneous Valvuloplasty-Mitral Valve |
| 6 | CS | M6532 | Percutaneous Valvuloplasty-Aortic Valve |
| 6 | CS | M6580 | Transcatheter Aortic Valve Implantation-Transapical Approach |
| 6 | CS | M6581 | Transcatheter Aortic Valve Implantation-Transaortic Approach |
| 6 | CS | M6582 | Transcatheter Aortic Valve Implantation-Transfemoral, Transsubclavian Approach |
| 6 | GS | O2056 | Thrombectomy (Artery),Others |
| 6 | GS | O2059 | Thrombectomy (Deep Vein),Others |
| 6 | NS | S4635 | Craniotomy for Excision of Brain Tumor-Supratentorial-Complex |
| 6 | NS | S4621 | Craniotomy for Evacuation of Hematoma-Subdural Or Extradural |
| 6 | NS | S4622 | Craniotomy for Evacuation of Hematoma-Intracerebral |
| 6 | NS | N0331 | Craniotomy or Craniectomy For Exploration |
| 6 | NS | N0334 | Craniotomy or Craniectomy For Excision of Cranial Lesion or Benign Tumor |
| 6 | URO | R3280 | Renal Transplantation |
| 7 | GS | Q7230 | Hepatopancreaticoduodenectomy |
| 7 | GS | Q2445 | Damage Control Laparotomy |
| 7 | CS | O0173 | Vascular Bypass Operation (Aorto to carotid and subclavian artery) |
| 7 | CS | O2034 | Resection of Aneurysm-Abdominal Aorta And Iliac Artery |
| 7 | CS | O1431 | Pneumonectomy |
| 7 | CS | Q2402 | Curative Operation of Esophageal Malignant Tumor-Thoracic And Abdominal Approach |
| 7 | CS | Q2403 | Curative Operation of Esophageal Malignant Tumor-Cervical, Thoracic And Abdominal Approach |
| 7 | CS | O1521 | Pericardiaolysis with Redo-sternotomy |
| 7 | CS | Q2362 | Esophageal Bypass Reconstruction-Thoracic And Abdominal Approach |
| 7 | CS | Q2366 | Esophageal Reconstruction After Resection-With Stomach |
| 7 | CS | Q2367 | Esophageal Reconstruction After Resection-With Jejunum |
| 7 | GS | O0224 | Resection of Aneurysm-Abdominal Aorta (Infrarenal) |
| 7 | OS | N0572 | Amputation of Extremities[Thigh] |
| 7 | CS | O1644 | Vascular Bypass Op (Aorta-Renal,Thoracic,Abdominal Aorta-Femoral,Aorta-Splanchnic),Artificial Vessel |
| 7 | CS | OA640 | Vascular Bypass Operation (Aorta-Coronary)-Simple (Off Pump CABG) |
| 7 | CS | OA641 | Vascular Bypass Operation (Aorta-Coronary),Simple |
| 7 | CS | OA648 | Vascular Bypass Operation (Aorta-Coronary)-Simple (Off Pump CABG) |
| 7 | CS | OA649 | Vascular Bypass Operation (Aorta-Coronary)-Simple (Off Pump CABG) |
| 7 | CS | O1640 | Vascular Bypass Operation (Aorta-Coronary)-Simple |
| 7 | CS | O1641 | Vascular Bypass Operation (Aorta-Coronary),Simple |
| 7 | CS | O1648 | Vascular Bypass Operation (Aorta-Coronary)-Simple |
| 7 | CS | O1649 | Vascular Bypass Operation (Aorta-Coronary)-Simple |
| 7 | CS | O1781 | Valvuloplasty-Tricuspid Valve |
| 7 | CS | O1782 | Valvuloplasty-Mitral Valve |
| 7 | CS | O1783 | Valvuloplasty-Aortic Valve |
| 7 | CS | O1791 | Valve Replacement-Tricuspid Valve |
| 7 | CS | O1792 | Valve Replacement-Mitral Valve |
| 7 | CS | O1793 | Valve Replacement-Aortic Valve |
| 7 | CS | O1799 | Sutureless Aortic Valve Replacement |
| 8 | CS | OA647 | Vascular Bypass Operation (Aorta-Coronary),Complex |
| 8 | CS | O1647 | Vascular Bypass Operation (Aorta-Coronary),Complex |
| 8 | CS | O1794 | Reoperation of Valvuloplasty-Tricuspid Valve |
| 8 | CS | O1795 | Reoperation of Valvuloplasty-Mitral Valve |
| 8 | CS | O1796 | Reoperation of Valvuloplasty-Aortic Valve |
| 8 | CS | O1823 | Left Ventricular Aneurysmectomy |
| 8 | CS | O1825 | Left Ventricular Outflow Track Augmentation |
| 8 | CS | O1950 | Pulmonary Artery Embolectomy |
| 8 | GS | Q8040 | Liver Transplantation from Cadaver Donor-Total |
| 8 | GS | O0223 | Resection of Aneurysm-Abdominal Aorta (Suprarenal[Juxtarenal]) |
| 8 | CS | O1722 | Operation of Ventricular Septal Defect-Post Infarction |
| 8 | CS | O2031 | Resection of Aneurysm-Ascending Aorta |
| 8 | CS | O2032 | Resection of Aneurysm-Aortic Arch |
| 8 | CS | O2033 | Resection of Aneurysm-Descending Thoracic Aorta |
| 8 | NS | N0333 | Craniotomy or Craniectomy For Decompression |

OG, Operation Group; GS, General Surgery; CS, Cardiothoracic Surgery; OS, Orthopedic surgery; URO, Urology; OPH, ophthalmic surgery; NS, Neuro-Surgery; ENT, Ear, Nose and Throat surgery; PS, Plastic Surgery; GY, Gynecologic surgery; DNT, Dental Surgery.

**References**

1. Gilbert T, Neuburger J, Kraindler J, Keeble E, Smith P, Ariti C, et al. Development and validation of a Hospital Frailty Risk Score focusing on older people in acute care settings using electronic hospital records: an observational study. Lancet 2018; 391: 1775-82.

2. Lee SW, Nam JS, Kim YJ, Kim MJ, Choi JH, Lee EH, et al. Predictive Model for the Assessment of Preoperative Frailty Risk in the Elderly. J Clin Med 2021; 10.

3. Lee SW, Kim KS, Park SW, Kim J, Choi JH, Lee S, et al. Application of the New Preoperative Frailty Risk Score in Elderly Patients Undergoing Emergency Surgery. Gerontology 2022: 1-9.
